# Integrated bioinformatics analysis identifies a ubiquitination-related gene signature in schizophrenia with peripheral clinical validation

**DOI:** 10.3389/fpsyt.2026.1987536

**Published:** 2026-10-29

**Authors:** Xiaojiao Bi, Bo Li, Mengyao Lv, Jieyu Yang, Dongdong Qiao

**Affiliations:** 1 Shandong Mental Health Center, Shandong University, Jinan, China; 2 The First Clinical College, Shandong University, Jinan, Shandong, China

**Keywords:** GEO, molecular signature, S100A8, schizophrenia, ubiquitination

## Abstract

**Background:**

Dysregulation of the ubiquitin proteasome pathway is implicated in the pathogenesis of schizophrenia (SCZ). The objective of this study is to identify the dysregulation of the ubiquitin-proteasome pathway and the potential mechanisms associated with SCZ by comprehensive analysis using integrated GEO datasets.

**Methods:**

We integrated three SCZ related datasets containing 1847 ubiquitination-related genes (URGs) from GeneCards and PubMed, addressing batch effects and normalization with R packages. Subsequent Gene Ontology enrichment analysis was applied to the identified URGs, and an 8-gene molecular signature was constructed to distinguish schizophrenia from control brain tissues, and the discriminatory performance was evaluated by the area under the Receiver Operating Characteristic curve. SCZ samples were categorized into high-risk and low-risk groups based on median Risk Scores, followed by gene set enrichment analysis.

**Results:**

A total of 13 SCZ-associated ubiquitin-related differentially expressed genes were significantly identified, with eleven being extremely statistically significant and two highly statistically significant. An 8-gene LASSO-derived molecular signature was constructed and validated, effectively discriminating SCZ patients from controls and classifying SCZ patients into high-risk and low-risk subgroups. The study also constructed protein-protein interaction and regulatory networks and conducted immune infiltration analysis, indicating statistically significant differences in six types of immune cells between the SCZ group and the control group, including CD56bright natural killer cells and central memory CD4+ T cells. ELISA validation in an independent clinical cohort confirmed elevated plasma S100A8 in SCZ patients versus controls, with no significant correlation to PANSS five-factor scores, suggesting S100A8 as a candidate peripheral biomarker reflecting disease state rather than current symptom severity.

**Conclusions:**

Together with the identification of 13 ubiquitination-related DEGs and an eight-gene LASSO signature demonstrating moderate discriminative accuracy for stratifying SCZ patients, this multi-faceted analysis reveals altered ubiquitination patterns in SCZ and provides a hypothesis-generating foundation for future prospective studies incorporating drug-naïve first-episode patients, longitudinal sampling, and appropriate disease controls.

## 1 Introduction

Schizophrenia (SCZ), a devastating neuropsychiatric disorder affecting 1% global population (1), imposes substantial socioeconomic burdens as a leading cause of disability worldwide. With 5-10% lifetime suicide risk (2, 3), its complex pathogenesis involves genetic-environmental interactions disrupting multiple neurotransmitter systems and neurodevelopmental signaling pathways (4, 5). Current pharmacological and psychological interventions remain suboptimal, particularly given antipsychotics’ limited efficacy and severe side effects (6). This therapeutic impasse underscores the critical need for identifying novel molecular biomarkers and targeted therapies to advance precision medicine approaches in SCZ management.

Ubiquitination, which regulates protein degradation, trafficking, and signaling through a complex machinery involving ubiquitin and various ligases, is crucial for protein homeostasis, DNA repair, cell cycle regulation, and immune responses (7). Emerging evidence has implicated dysregulation of the ubiquitin-proteasome system in SCZ pathogenesis (8–10). Luza et al. identified specific UPS component abnormalities in SCZ, including UBA6, UBE3B, Parkin, and FBXL21 (5). However, as observed in many psychiatric ubiquitination studies, these candidate biomarkers frequently lack reproducibility. This underscores the need for comprehensive investigations of ubiquitination networks in SCZ. Our study addresses this gap through integrated analysis of GEO datasets to identify SCZ-associated ubiquitin-related differentially expressed genes (URDEGs).

In this study, batch effect removal was executed based on three GEO datasets, followed by differential expression analysis, as well as a combination of analyses using multiple methods such as Principal Component Analysis (PCA), Receiver Operating Characteristic (ROC) curve, Gene Ontology (GO) enrichment analysis and Gene Set Enrichment Analysis (GSEA). The advantage of this is that multiple datasets could be considered in an integrated manner, improving the reliability and accuracy of the results. The aim of this study is to identify important genes associated with SCZ and their potential mechanisms, and to provide new ideas for future mechanistic research and biomarker development. Beyond transcriptomic bioinformatic screening across multiple cohorts, we performed ELISA measurements on independent clinical plasma samples to validate S100A8, the core candidate biomarker. We also examined its associations with psychotic symptoms, aiming to bridge these computational findings to clinical observations.

## 2 Materials and methods

### 2.1 Data download

Three SCZ-associated microarray datasets (GSE21138 (11), GSE21935 (11), GSE12649 (12)) were retrieved from the GEO database (13) via GEOquery (14) (v2.70.0). All human brain-derived datasets included: GSE21138 (GPL570, 30 SCZ/29 controls), GSE21935 (GPL570, 23 SCZ/19 controls), and GSE12649 (GPL96, 35 SCZ/34 controls) (**Supplementary Table S1**). Ubiquitin-related genes (URGs) were curated from GeneCards (15) (“Ubiquitin” search, score >4; n=1,847) supplemented by 11 PubMed (16)-derived URGs, yielding 1847 non-redundant URGs. The sva (17) package (v3.50.0) integrated datasets into a combined cohort (88 SCZ/82 controls). Limma (18) (v3.58.1)processed data through normalization, probe annotation, and batch correction. Principal component analysis (PCA) (19) validated batch effect removal by comparing pre-/post-processing expression matrices in reduced-dimensional space.

### 2.2 Related ubiquitin change differentially expressed genes related to SCZ

According to the sample grouping of the integrated GEO datasets (Combined Datasets), samples were divided into a schizophrenia (SZ) group and a control group. Differential expression analysis of genes between the SZ and control groups was performed using the R package limma (18)(version 3.58.1). The thresholds for identifying differentially expressed genes (DEGs) were set as |logFC| > 0 and adjusted p < 0.05. Genes with logFC > 0 and adjusted p < 0.05 were defined as up-regulated DEGs, whereas genes with logFC < 0 and adjusted p < 0.05 were defined as down-regulated DEGs. P values were adjusted using the Benjamini–Hochberg (BH) method. The results of the differential expression analysis were visualized as a volcano plot using the R package ggplot2 (version 3.4.4).

To obtain ubiquitin-related differentially expressed genes (URDEGs) associated with schizophrenia (SZ), all DEGs with |logFC| > 0 and adjusted p < 0.05 identified from the integrated GEO datasets (Combined Datasets) were intersected with the ubiquitin-related genes (URGs), and the overlap was visualized with a Venn diagram. The resulting URDEGs were then displayed in a heatmap generated with the R package pheatmap (version 1.0.12).

### 2.3 Gene ontology analysis

GO analysis (20) is a common method to conduct large-scale functional enrichment studies, including Biological Process (BP), Cell Component (CC), and Molecular Function (MF). The R package clusterProfiler (21) (Version 4.10.0) was employed for URGs enrichment in GO analysis, with an entry selection criteria for value *P* < 0.05.

### 2.4 Construction of molecular signature for SCZ

To construct a diagnostic model for schizophrenia (SZ) based on the integrated GEO datasets (Combined Datasets), logistic regression was first applied to the ubiquitin-related differentially expressed genes (URDEGs) to examine their association with sample group, a binary variable indicating SZ or control samples. URDEGs with p < 0.05 were retained to construct the logistic regression model, and the group-specific expression of the URDEGs included in the model was visualized with a forest plot.

Subsequently, a support vector machine (SVM) model was constructed based on the URDEGs retained in the logistic regression mode (22), and the optimal number of URDEGs was determined according to the highest accuracy and lowest error rate.

Finally, least absolute shrinkage and selection operator (LASSO) regression was performed on the URDEGs retained in the SVM model using the R package glmnet (version 4.1-8) (23), with parameters set as set.seed(500) and family = “binomial”. LASSO regression reduces overfitting and improves model generalizability by adding a penalty term (lambda × the absolute value of the coefficient) to the linear regression framework. To further prevent overfitting, ten-fold cross-validation was applied (cv.glmnet, family = “binomial”, type.measure = “class”), and the optimal penalty parameter (lambda) was determined using the minimum criterion (lambda.min). The genes with non-zero coefficients at lambda.min constituted the final 8-gene diagnostic model, and the LASSO regression results were visualized with a coefficient trajectory plot. The genes included in the final model were defined as model genes (Model Genes), and the LASSO risk score (RiskScore) was calculated based on their regression coefficients using the following formula:


$$riskScore=\sum_{i}Coefficient\left(gene_{1}\right)*mRNA\;Expression\left(gene_{1}\right)$$

In actual analysis and calculation, eight genes were involved in the computation. The formula is as follows.


$$\begin{array}{c} riskScore=KPNB1ZFRS**\left(4.076\right)+\left(1.965\right)+\left(3.174\right)+MLF1UBR5*\\ {}*\left(1.462\right)\left(1.460\right)++PAK2*S100A8KDM5A**\left(0.253\right)+\\ +\left(1.703\right)ETV5*\left(0.144\right) \end{array}$$

### 2.5 Validation of the SCZ molecular signature

First, receiver operating characteristic (ROC) curves were generated for the Combined GEO Datasets using the R package pROC (24) (v1.18.5) to calculate the Area Under the Curve (AUC). Subsequently, a nomogram (25) was constructed via the R package rms (v6.7-1) to visualize relationships between signature genes using logistic regression coefficients. Calibration plots derived from LASSO regression analysis assessed the predictive accuracy of the molecular signature, while decision curve analysis (DCA) evaluated clinical utility. DCA curves were generated using the ggDCA package (v1.1) based on signature genes in the Combined GEO Datasets (26).

### 2.6 Friends analysis

The semantic comparison of GO (20) annotation provides a quantitative method for calculating the similarity between genes and genomes, and has become an important basis for many bioinformatics analysis methods. Functional similarity Friends analysis of signature genes was performed by R package GOSemSim (27) (Version 2.28.0).

### 2.7 Expression validation and discriminatory performance analysis by ROC curve

To investigate expression differences of URDEGs and signature genes between SCZ and control samples in the Combined GEO Datasets, group comparison plots were generated. The R package pROC (24) (v1.18.5) was employed to construct ROC curves and calculate AUC values, where AUC ranges (0.5-1.0) reflect discriminatory capacity: 0.5-0.7 = low, 0.7-0.9 = moderate, >0.9 = high accuracy. SCZ samples were stratified into high/low-risk groups using median RiskScore values. Differential expression patterns of signature genes between risk groups were visualized through comparative plots. ROC analysis was repeated for signature genes (24) to assess their discriminatory performance for SCZ onset, maintaining consistent AUC interpretation criteria.

### 2.8 Gene set enrichment analysis

GSEA (28) analyzed Combined GEO Datasets using clusterProfiler (v4.10.0) with parameters: seed=2020, 10,000 permutations, gene sets (10-500 genes) from the Molecular Signatures Database (MSigDB) (29) c2 pathways (6,449 entries). SCZ samples were stratified by RiskScore medians into high-/low-risk groups. Limma (v3.58.1) identified DEGs (|logFC|>0, FDR p<0.05), visualized via volcano plots (ggplot2) and heatmaps (pheatmap). GSEA of all samples under identical parameters revealed pathways (adjusted p<0.05).

### 2.9 Construction of protein interaction and regulatory network

Using the STRING database (30), we constructed a Protein-Protein Interaction (PPI) network for signature genes with an interaction confidence threshold>0.150 (low stringency). Highly interconnected subnetworks were prioritized as potential functional complexes, with hub genes selected for downstream analysis. To investigate transcriptional regulation, transcription factors (TFs) interacting with signature genes were identified via the ChIPBase database (31). Cytoscape (32) visualized TF-mRNA regulatory networks, revealing transcriptional regulation mechanisms. Concurrently, miRNA-mRNA interactions were analyzed using StarBase v3.0 (33), which catalogs experimentally validated miRNA-target relationships. The resulting mRNA-miRNA network, also mapped in Cytoscape (32), identified multi-target regulatory patterns where single miRNAs regulate multiple genes and vice versa.

### 2.10 Immune analysis

Single-sample Gene Set Enrichment Analysis (ssGSEA) (34) quantified immune infiltration in Combined GEO Datasets across 28 subtypes (e.g., Activated CD8+ T cells). Enrichment scores (ggplot2 v3.4.4) compared SCZ vs control distributions (p<0.05 retained). Spearman correlations (pheatmap v1.0.12) mapped immune subtype interrelationships and signature gene-immune interactions (bubble plots, p<0.05). Reapplied to HighRisk/LowRisk-stratified SCZ samples, comparative plots revealed risk-associated immune disparities, while heatmaps delineated intra-group subtype correlations. Layered bubble plots contrasted risk-specific gene-immune regulatory patterns.

### 2.11 Statistical analysis

Bioinformatic analyses were performed in R v4.3.0, while the clinical analyses of plasma S100A8 and PANSS scores were performed in Python v3.12.9. Two-group comparisons employed Student’s t-test (normal variables) or Wilcoxon rank-sum test (non-normal). Multi-group comparisons used Kruskal-Wallis. Spearman correlation quantified molecular relationships. Unless specified, all p-values were two-tailed, with p<0.05 considered statistically significant.

### 2.12 ELISA detection of plasma S100A8

This study was conducted in accordance with the Declaration of Helsinki and approved by the Ethics Committee of Shandong Mental Health Center (approval No. 2021-[21], dated March 1, 2021). An independent clinical cohort was employed to verify the peripheral expression of S100A8. The cohort included 33 schizophrenia patients and 35 healthy controls matched for age and gender. All patients were diagnosed based on the Diagnostic and Statistical Manual of Mental Disorders (DSM-V). Subjects with severe systemic diseases, malignant tumor history, participation in other interfering clinical trials, or unwillingness to provide informed consent were excluded, as well as those deemed inappropriate for enrollment by researchers. All participants signed informed consent, and the study protocol was approved by the local ethics committee.

Fasting peripheral venous blood samples were collected from all participants. Plasma was separated by centrifugation and stored at −80 °C for subsequent detection. Plasma S100A8 protein levels were measured using Human S100A8 (S100 Calcium Binding Protein A8) ELISA Kit (Elabscience^®^, Cat#E-EL-H6275), following the manufacturer’s instructions.

### 2.13 PANSS five-factor scoring and clinical association analysis

PANSS symptom dimensions were calculated using the Wallwork consensus five-factor model (35). The positive factor included P1, P3, P5, and G9; the negative factor included N1, N2, N3, N4, N6, and G7; the disorganized/cognitive factor included P2, N5, and G11; the excited factor included P4, P7, G8, and G14; and the depressed factor included G2, G3, and G6. The score for each factor was calculated as the mean of its constituent items. The five-factor structure has also been supported by a meta-analysis and validated in a Chinese Mandarin version of the PANSS (36, 37). Two-sided Spearman correlations were used to examine the relationships between plasma S100A8 and the five PANSS factor scores. A total of 20,000 bootstrap resamples at the participant level were used to calculate 95% confidence intervals, and 99,999 permutations were used to calculate P values. The P values for these five analyses were corrected using the Benjamini-Hochberg false discovery rate. The associations of age with plasma S100A8 and each PANSS factor were examined using Spearman correlation. Differences between men and women were analyzed using the two-sided Mann-Whitney U test. Partial Spearman correlations were then used to examine the associations between plasma S100A8 and PANSS factor scores after adjustment for age and sex. The Benjamini-Hochberg method was also applied to the five adjusted analyses.

## 3 Results

### 3.1 Screening for SCZ associated URDEGs

According to the technology roadmap (**Supplementary Figure S1**), We obtained SCZ associated URDEGs. Firstly, we consolidated SCZ-related GEO datasets (GSE21138, GSE21935, GSE12649) using the R package sva to eliminate batch effects (**Supplementary Figure S2**). This process was validated through two complementary approaches: (1) Distribution boxplots (**Supplementary Figures S2A, B**) demonstrated significant reduction in expression value discrepancies between original and batch-corrected datasets, and (2) Principal component analysis (PCA) plots (**Supplementary Figures S2C, D**) revealed substantially improved low-dimensional clustering consistency post-batch correction. Subsequently, differential gene expression analysis was performed using limma on the integrated GEO datasets, identifying 123 differentially expressed genes (DEGs) with |logFC| > 0 and adjusted P-value < 0.05. Among these, 82 were upregulated and 41 downregulated (**Figure 1A**). To identify ubiquitin-related differentially expressed genes (URDEGs), we intersected DEGs with ubiquitin-related genes (URGs) through Venn diagram analysis (**Figure 1B**), yielding 13 URDEGs (**Supplementary Table S2**). Finally, integrative group analysis confirmed these URDEGs’ differential expression patterns across sample groups, which were visualized through a heat map generated by pheatmap (**Figure 1C**).

**Figure 1:**
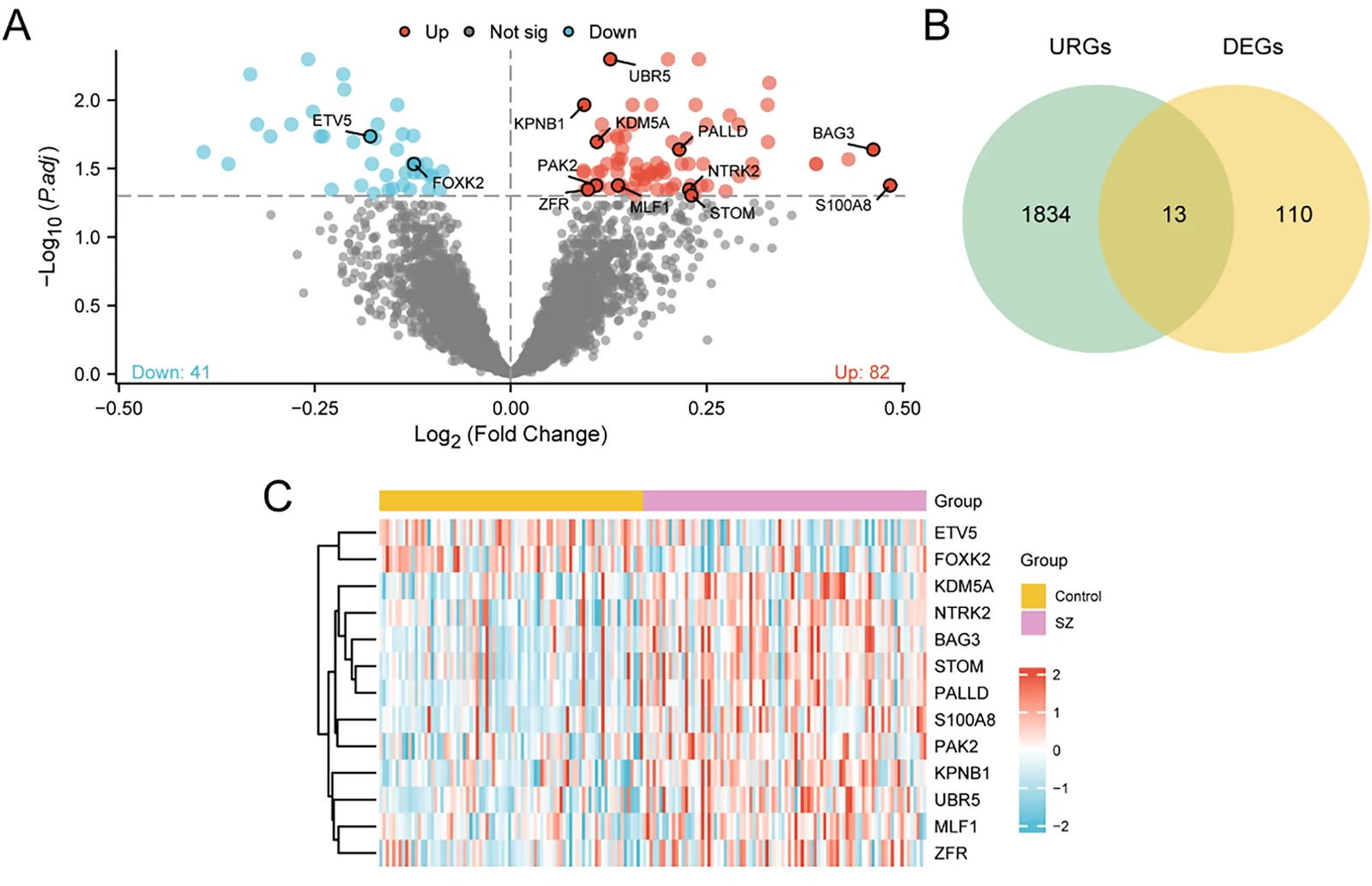
Differential gene expression analysis. **(A)** Volcano plot of differentially expressed gene analysis between SCZ and Control groups in Combined GEO Datasets. **(B)** integrated GEO data set of DEGs and URGs Venn figure. **(C)** Heat map of URDEGs in the integrated GEO Datasets. SCZ, Schizophrenia; DEGs, Differentially Expressed Genes; URGs, Ubiquitin-Related Genes; URDEGs, Ubiquitin-Related Differentially Expressed Genes. Pink is the SCZ group and yellow is the Control group. Red represents high expression in heat map, blue represents lower expression.

### 3.2 Differential expression verification and ROC curve analysis

To explore the expression differences of URDEGs in Combined GEO Datasets, the group comparison figure (**Figure 2A**) shows the difference in expression of URDEGs between SCZ and control samples in the integrated GEO Datasets. The differential results indicate that the expression levels of 11 URDEGs (*UBR5, KPNB1, KDM5A, BAG3, PALLD, MLF1, S100A8, NTRK2, STOM, ETV5, FOXK2*) are highly statistically significant between SCZ and control group samples (p value<0.001). There are significant statistical differences in the expression levels of *PAK2* and *ZFR* between SCZ and control samples (p value < 0.01) in the combined datasets. Finally, using the R package pROC to plot ROC curves based on the expression levels of URDEGs in the combined GEO dataset. The ROC curves (**Figures 2B–G**) indicated that the expression levels of 11 URDEGs (*KPNB1*, *KDM5A*, *BAG3*, *MLF1*, *PAK2*, *S100A8*, *NTRK2*, *ZFR*, *STOM*, *ETV5*, and *FOXK2*) had low discriminative accuracy (0.5 < AUC < 0.7) between SCZ and control samples, whereas the expression levels of two URDEGs (*UBR5*, *PALLD*) achieved moderate accuracy (0.7 < AUC < 0.9).

**Figure 2:**
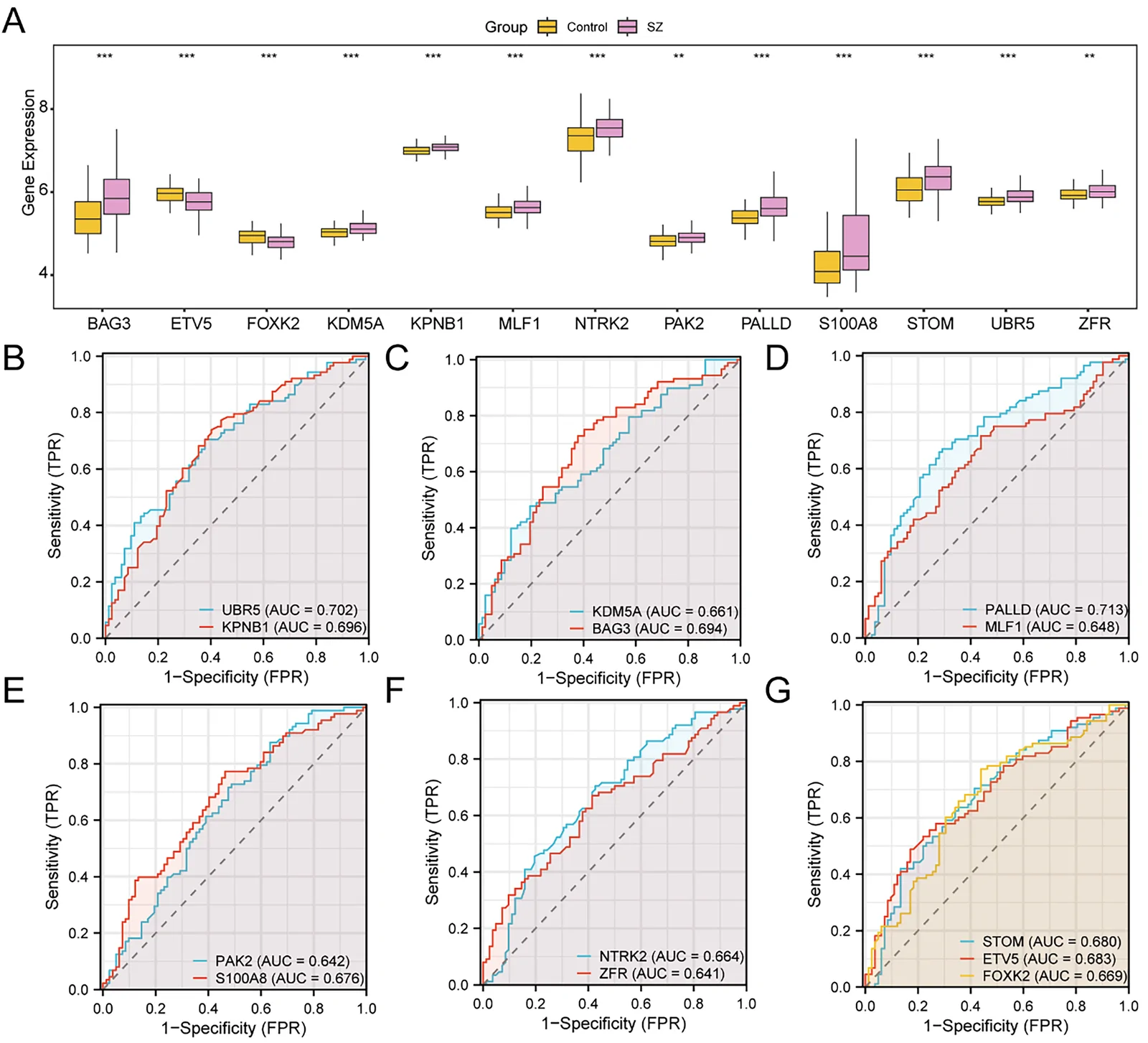
Differential Expression Validation and Correlation Analysis. **(A)**. URDEGs in the integration of GEO data set SCZ and Control group comparison chart in the sample. B-F.ROC curves of URDEGs in Combined GEO Datasets. *UBR5* and *KPNB1* **(B)**, *KDM5A* and *BAG3* **(C)**, *PALLD* and *MLF1* **(D)**, *PAK2* and *S100A8* **(E)**, *NTRK2* and *ZFR*S **(F)**, *STOM*, *ETV5* and *FOXK2* **(G)**. Grouping comparison chart in yellow for Control group, pink on behalf of SCZ group. ** on behalf of the p value<0.01, highly statistically significant; ***represents p value<0.001 and highly statistically significant.

### 3.3 Gene ontology enrichment and pathway analysis

To investigate the biological significance of the 13 identified URDEGs, we performed systematic GO enrichment analysis focusing on biological processes (BP), cellular components (CC), and molecular functions (MF). The analysis revealed significant enrichment in critical pathways: Protein import into nucleus and Nuclear transport (BP), Stress fiber and Actin filament bundle (CC), and Small GTPase binding and Protein serine/threonine kinase activity (MF) (**Supplementary Table S3**; **Supplementary Figure S3A**). Visualization through histogram plots confirmed hierarchical clustering of enriched terms, with nodes representing higher-order biological concepts containing more constituent molecules (**Supplementary Figures S3B–D**). This analysis was complemented by Gene Set Enrichment Analysis (GSEA) of the integrated GEO datasets to evaluate global transcriptional signatures associated with SCZ. GSEA identified robust enrichment in pathways including Oxidative Stress Response Up, Lysosome, Energy Dependent Regulation Of MTOR By Lkb1/Ampk, and Clathrin Mediated Endocytosis (**Supplementary Figures S4A–E**; **Supplementary Table S4**). Notably, these findings highlight convergent molecular mechanisms involving cellular stress responses, lysosomal function, and metabolic regulation, which are critical for understanding SCZ pathophysiology.

### 3.4 Construction of molecular signature for SCZ

First of all, in order to determine 13 URDEGs related to discriminatory performance in SCZ, based on 13 URDEGs related to (Logistic regression) and build logic (Logistic regression model) and through the Forest figure (Forest Plot) of visual display (**Figure 3A**). The results showed that 13 URDEGs were statistically significant in the Logistic regression model (p value < 0.05). Then, based on 13 URDEGs related to and SVM (Support Vector Machine) algorithm to construct the SVM model, and get the lowest error rate (**Figure 3B**), and the highest accuracy (**Figure 3C**), the number of genes. The result shows that when the number of genes is 9, the accuracy of the SVM model is highest. Then, the LASSO regression analysis was performed to construct a molecular signature based on the 9 URDEGs included in SVM model. By mapping the LASSO regression model (**Figure 3D**) and LASSO variable trajectory diagram (**Figure 3E**) of visual display. Results show that the LASSO regression Model contains eight URDEGs signature genes, respectively is: *KPNB1*, *ZFR*S, *UBR5*, *MLF1*, *PAK2*, *S100A8*, *KDM5A*, *ETV5*.

**Figure 3:**
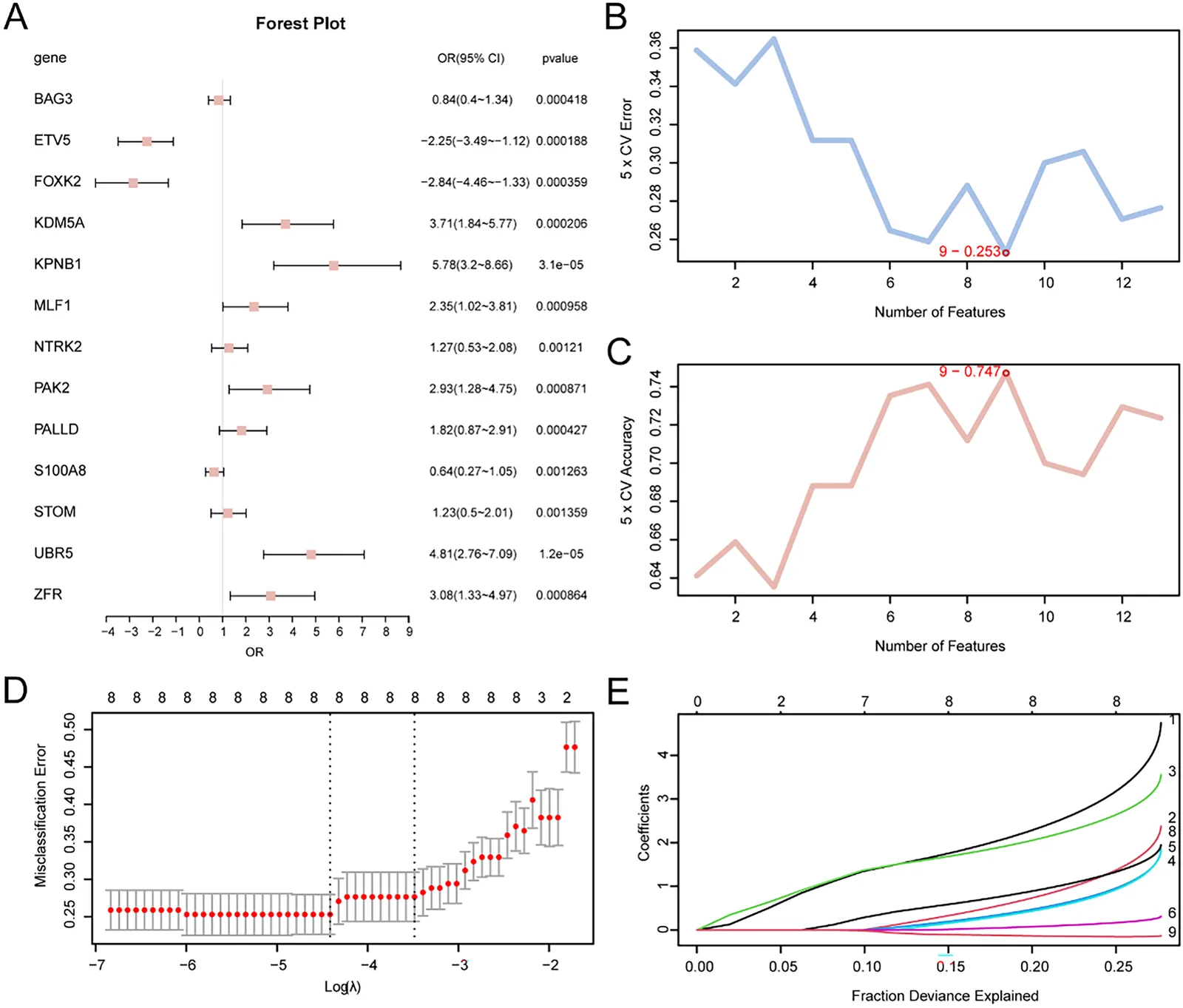
Diagnostic Model of SCZ. **(A)** A Forest Plot of 13 URDEGs within the SCZ diagnostic model based on the Logistic regression model. **(B, C)** The number of genes with the lowest error rate **(B)** and the number of genes with the highest accuracy **(C)** obtained by the SVM algorithm are visualized. **(D, E)** Diagnostic model plot **(D)** and variable trajectory plot **(E)** of LASSO regression model. URDEGs: Ubiquitin-Related Differentially Expressed Genes; SCZ, schizophrenia; SVM, Support Vector Machine; LASSO, Least Absolute Shrinkage and Selection Operator.

### 3.5 Validation and functional analysis of the SCZ molecular signature

We evaluated the SCZ molecular signature using the R package pROC. The ROC curve analysis based on risk scores demonstrated moderate-to-high discriminative accuracy (AUC = 0.71–0.89) across the integrated GEO datasets (**Figure 4A**). A nomogram constructed from signature genes revealed that *KPNB1* exhibited significantly higher discriminatory utility compared to other variables, while *ETV*5 showed notably lower utility (**Figure 4B**). Calibration curve analysis indicated near-optimal prediction performance, with the model’s predictions closely aligning with observed probabilities despite slight deviations from the ideal diagonal line (**Figure 4C**). DCA supported the applicability of the signature for molecular discrimination across diverse subgroups, maintaining stability above all-negative and all-positive thresholds (**Figure 4D**). Finally, functional similarity analysis (Friends) prioritized *KPNB1* as a critical hub gene in SCZ pathogenesis, with its similarity score approaching the critical threshold (cut-off = 0.7; **Figure 4E**). These results collectively establish the model’s validity and highlight *KPNB1*’s central role in SCZ molecular mechanisms.

**Figure 4:**
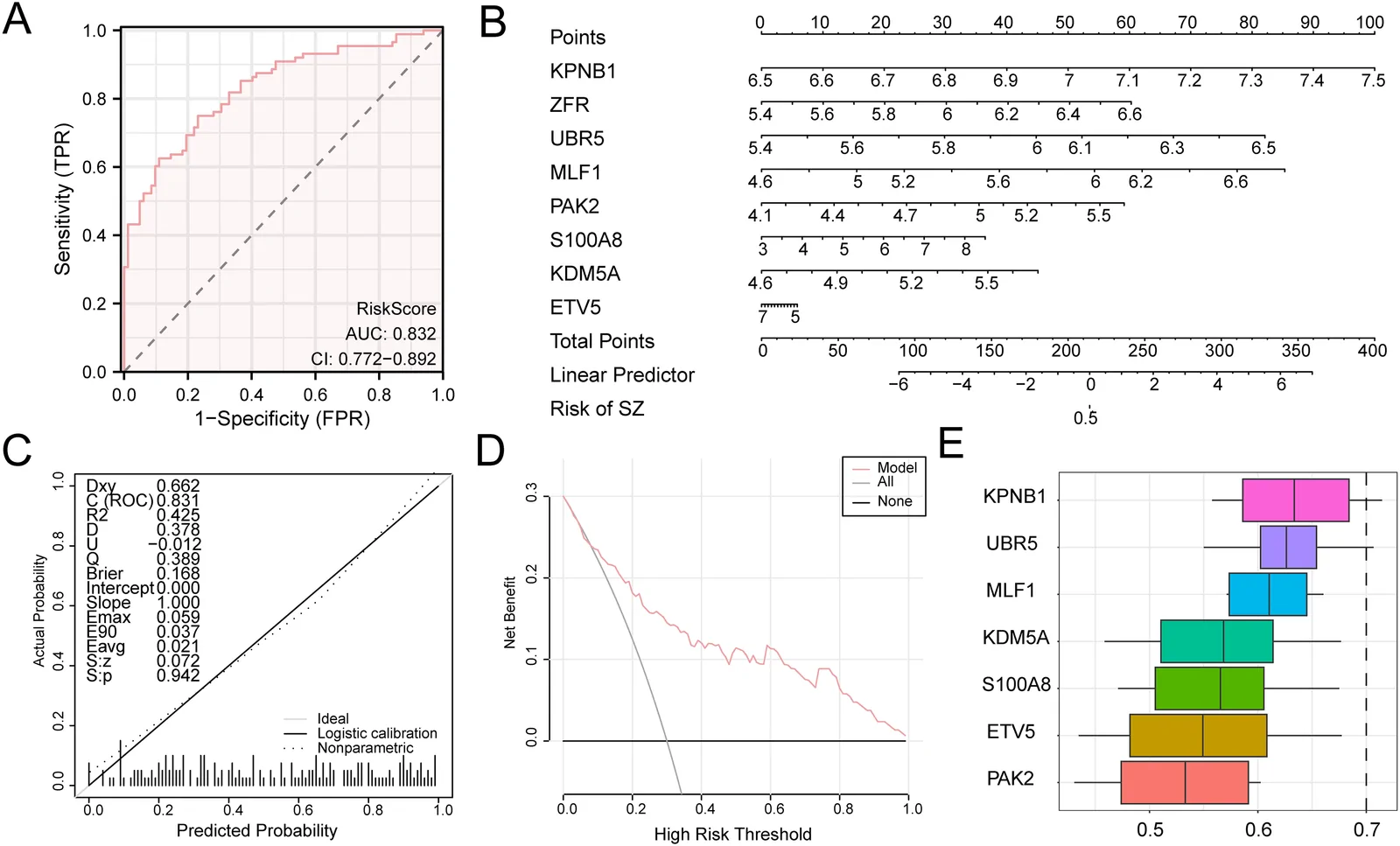
Diagnostic and Validation of SCZ and Friends Analysis. **(A)**. ROC curves of RiskScore in the Combined GEO Datasets. **(B)** Model Genes in SCZ diagnosis Model of integration of GEO data set columns in the chart (Nomogram). **(C, D)** SCZ diagnosis Model based on integration of GEO data set Model Genes of the Calibration Curve **(C)** and DCA figure **(D)**. **(E)** The function of Model Genes similarity Friends analysis results. The vertical axis of the DCA plot represents net benefit, while the horizontal axis represents the probability threshold or threshold probability. The AUC of the ROC curve is generally between 0.5 and 1. The closer the AUC is to 1, the better the diagnostic performance. The AUC has some accuracy in the range of 0.7 to 0.9. ROC, receiver operating characteristic; SCZ, schizophrenia; GEO, Gene Expression Omnibus; DCA, decision curve analysis; AUC, Area Under the Curve.

### 3.6 Expression verification and discriminatory performance analysis of signature genes

Based on median risk scores, SCZ samples were stratified into high-risk and low-risk groups. Differential expression analysis of eight signature genes revealed significant group differences (**Figure 5A**). Five genes (KPNB1, UBR5, MLF1, S100A8, ETV5) exhibited highly significant differential expression (p< 0.001), with KPNB1 and ETV5 showing the largest fold changes. Two additional genes (KDM5A, ZFR) demonstrated moderate significance (p< 0.01), while PAK2 showed no significant difference (p> 0.05). The ROC curves (**Figures 5B–D**) showed that the expression levels of five signature genes (KPNB1, UBR5, MLF1, S100A8, and ETV5) achieved moderate accuracy (0.7 < AUC < 0.9) in discriminating between the high-risk and low-risk groups, while the remaining three signature genes (PAK2, KDM5A, and ZFR) showed low accuracy (0.5 < AUC < 0.7).These findings establish a robust molecular signature for SCZ risk stratification, with KPNB1 emerging as a critical biomarker.

**Figure 5:**
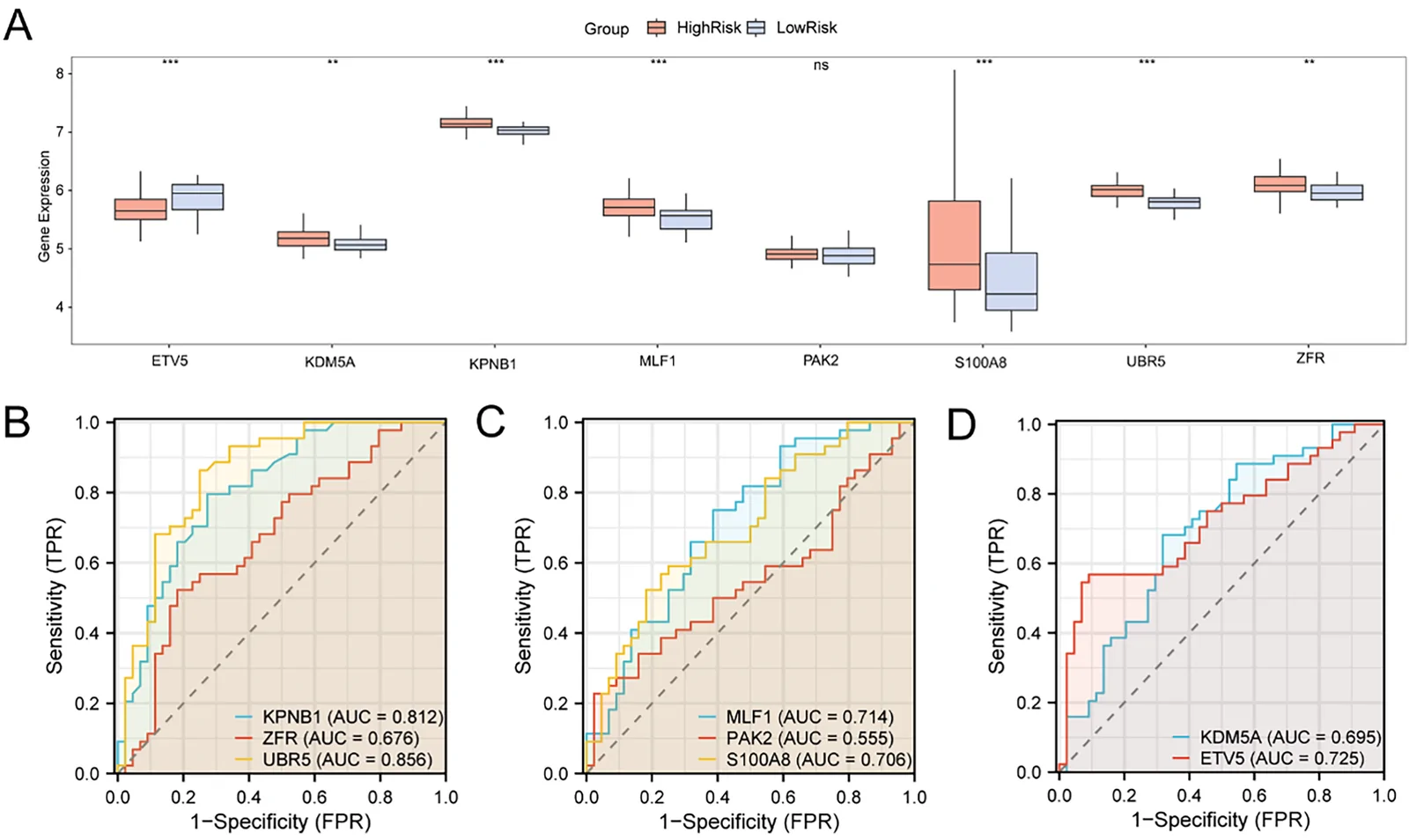
Differential expression validation and correlation analysis. **(A)** Group comparison diagram of Model Genes in the High Risk group and the Low Risk group of SCZ samples. B.-D Model Genes *KPNB1*, *ZFR*, *UBR5* **(B)**, *MLF1*, *PAK2*, *S100A8* **(C)**, *KDM5A*, *ETV5* **(D)** in the integration of GEO data set SCZ samples of ROC curve. Grouping comparison chart of shallow red stands for High Risk group (High Risk), light blue represents the Low Risk group. ns represents p value ≥ 0.05, no statistical significance; **represents p value < 0.01, highly statistically significant; ***represents p value < 0.001 and highly statistically significant. When AUC > 0.5, it indicates that the expression of the molecule is a trend to promote the occurrence of the event, and the closer the AUC is to 1, the better the diagnostic effect. AUC in 0.5 - 0.7 with low accuracy, AUC in 0.7 - 0.9 there is a certain accuracy.

### 3.7 GSEA of high and low risk group

Based on median risk scores, SCZ samples were stratified into high-risk and low-risk groups. Using limma, differential expression analysis identified 694 DEGs in the integrated GEO datasets: 337 upregulated (logFC > 0, adjusted P-value < 0.05) and 357 downregulated (logFC < 0, adjusted P-value < 0.05), as visualized by volcano plot (**Supplementary Figure S5A**). Top 10 DEGs were hierarchically clustered using pheatmap (**Supplementary Figure S5B**). GSEA revealed significant enrichment of biological processes and pathways in high-risk versus low-risk groups. Notably, Oxidative Stress Response Up (**Supplementary Figure S5D**), Oxidative Stress By Alexandra Bohne & Tbh (**Supplementary Figure S5E**), Oxidative Stress Response (**Supplementary Figure S5F**), and PI3K-AKT Signaling Pathway (**Supplementary Figure S5G**) were prominent among the top 20 enriched terms (**Supplementary Table S5**). These findings highlight dysregulation of oxidative stress and metabolic pathways in SCZ pathogenesis, with high-risk samples showing consistent molecular signatures across these networks.

### 3.8 Construction of PPI and gene regulatory networks

First of all, analysis of PPI, using the STRING database construction of eight signature gene of PPI Network (**Supplementary Figure S6A**). The results of the PPI network indicate that there are interactions among the five signature genes. Secondly, through the StarBase v3.0 gene database access and signature genes associated microRNAs, The mRNA-miRNA Regulatory Network was constructed and visualized by Cytoscape software (**Supplementary Figure S6B**). Among them, there were 3 signature genes and 20 mRNAs. Then, obtaining TFs interacting with signature genes from the ChIPBase database, an mRNA-TF regulatory network was constructed and visualized using Cytoscape software (**Supplementary Figure S6C**). This network includes 6 signature genes and 16 TFs, with detailed information provided in **Supplementary Table S6**.

### 3.9 Immune infiltration analysis in SCZ

Using ssGSEA, we quantified 28 immune cell abundances in SCZ samples. Group comparison analysis identified six immune cell types with significant differential infiltration between SCZ and control groups (p < 0.05): CD56 bright natural killer cells, central memory CD4+ T cells, eosinophils, γδ T cells, MDSCs, and T follicular helper cells (**Figure 6A**). Correlation analysis revealed strong positive associations (r=0.613, p<0.05) between MDSCs and central memory CD4+ T cells, while eosinophils and CD56 bright natural killer cells showed significant negative correlations (r=0.35, p<0.05) (**Figure 6B**). Finally, a bubble plot demonstrated that *S100A8* exhibited strong positive correlation with MDSCs (r=0.708, p<0.05), and *ETV5* showed significant negative correlation with central memory CD4+ T cells (r = 0.435, p < 0.05) (**Figure 6C**). These findings highlight immune cell dysregulation in SCZ pathogenesis.

**Figure 6:**
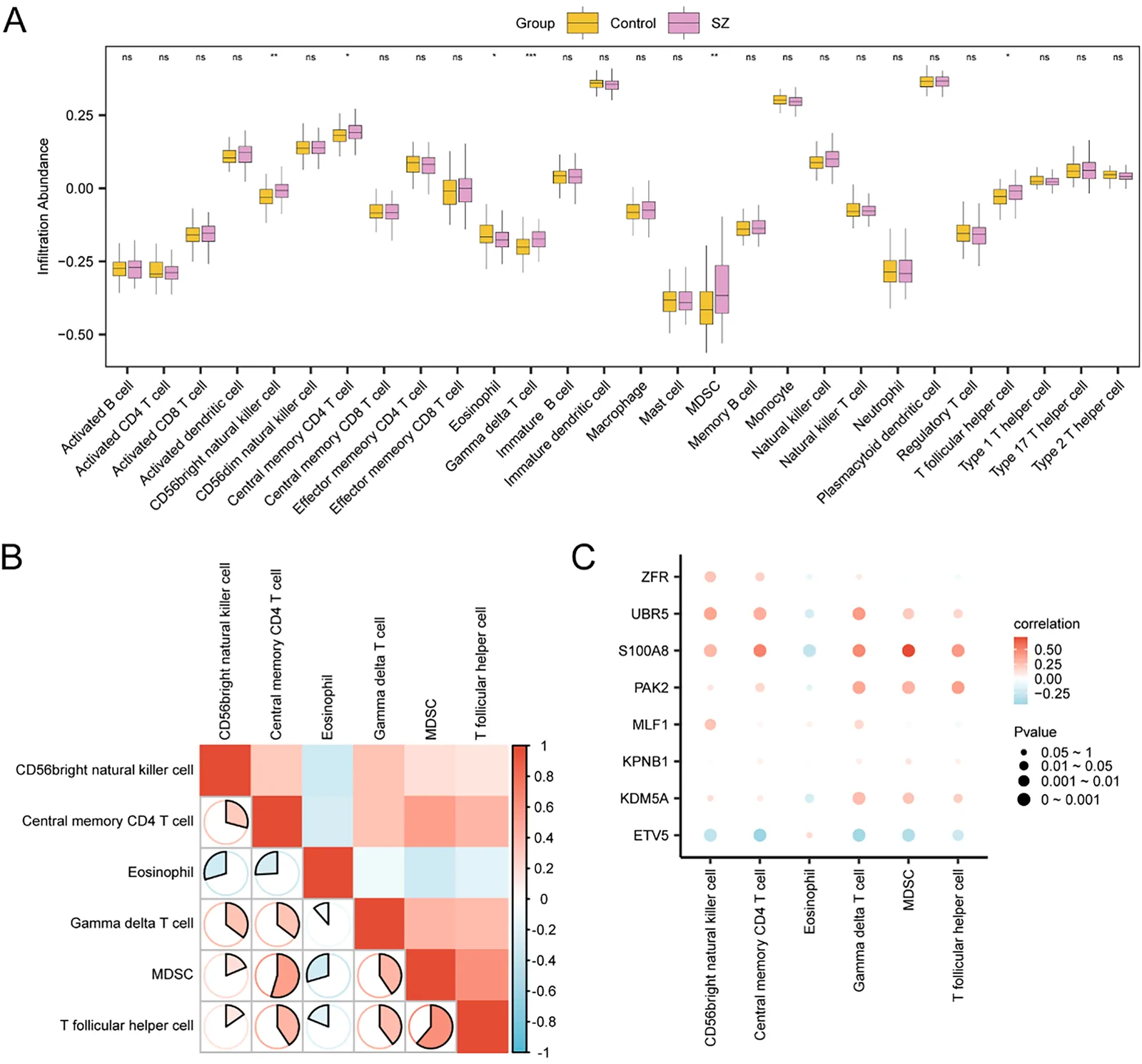
Combined datasets immune infiltration analysis by ssGSEA algorithm. **(A)** the immune cells in the integration of GEO data set the grouping comparison chart. **(B)** the immune cells in the integration of GEO data set (Combined Datasets) correlation of heat maps. **(C)** Correlation bubble plots of immune cell infiltration abundance and Model Genes in the Combined GEO Datasets. Ns on behalf of the p value of 0.05 or higher, no statistical significance; *represents p value < 0.05, statistically significant; **represents p value < 0.01, highly statistically significant; ***represents p value < 0.001 and highly statistically significant. The absolute value of correlation coefficient (r value) below 0.3 was weak or no correlation, between 0.3 and 0.5 was weak correlation, and between 0.5 and 0.8 was moderate correlation. Yellow is Control group, pink is SCZ group. Red indicates positive correlation and blue indicates negative correlation. The depth of the color represents the strength of the correlation.

### 3.10 High and low risk group of immune infiltration analysis

Using ssGSEA, we analyzed immune cell infiltration in SCZ high-risk samples via 28 immune cell profiles. Group comparison (**Supplementary Figure S7A**) identified six significantly altered cell types (p<0.05): CD56 bright natural killer cells, central memory CD4+ T cells, γδ T cells, macrophages, myeloid-derived suppressor cells (MDSCs), and monocytes. Correlation analysis (**Supplementary Figures S7B, C**) revealed strong intra-group positive correlations among most immune cells in high-risk SCZ samples. Bubble plot analysis (**Supplementary Figures S7D, E**) demonstrated significant associations: *S100A8* positively correlated with MDSCs (r=0.678, p<0.05), while low-risk group *KPNB1* showed positive correlation with CD56 bright natural killer cells (r=0.34, p<0.05). These findings highlight immune network abnormalities in SCZ pathogenesis.

### 3.11 Plasma S100A8 quantification and correlation analysis

In the transcriptomic bioinformatics analyses, we screened out S100A8 as one of the core immune-related biomarkers with dysregulated expression. Notably, its protein stability is directly governed by the ubiquitin-proteasome pathway (38, 39), aligning with the core focus of this study. Distinct from other intracellular or membrane-located candidate genes identified in this study, S100A8 is a typical secreted calcium-binding protein. It can be actively released into the peripheral bloodstream under inflammatory and pathological states, providing a reliable molecular basis for peripheral plasma detection. To further verify its abnormal expression and explore its clinical translational potential in SCZ, we adopted the ELISA method for validation using independent clinical samples.

The validation cohort consisted of 33 patients clinically diagnosed with SCZ and 35 healthy controls (HCs) matched for age and gender. Consistent with the upregulated mRNA expression of S100A8 in SCZ brain tissues observed in integrated GEO datasets (**Figure 2A**), ELISA results showed that plasma S100A8 protein concentrations were significantly higher in the SCZ group than in healthy controls (*P* < 0.05) (**Figure 7**). This peripheral protein expression pattern was highly consistent with transcriptomic alterations, confirming the aberrant expression of S100A8 in SCZ.

**Figure 7:**
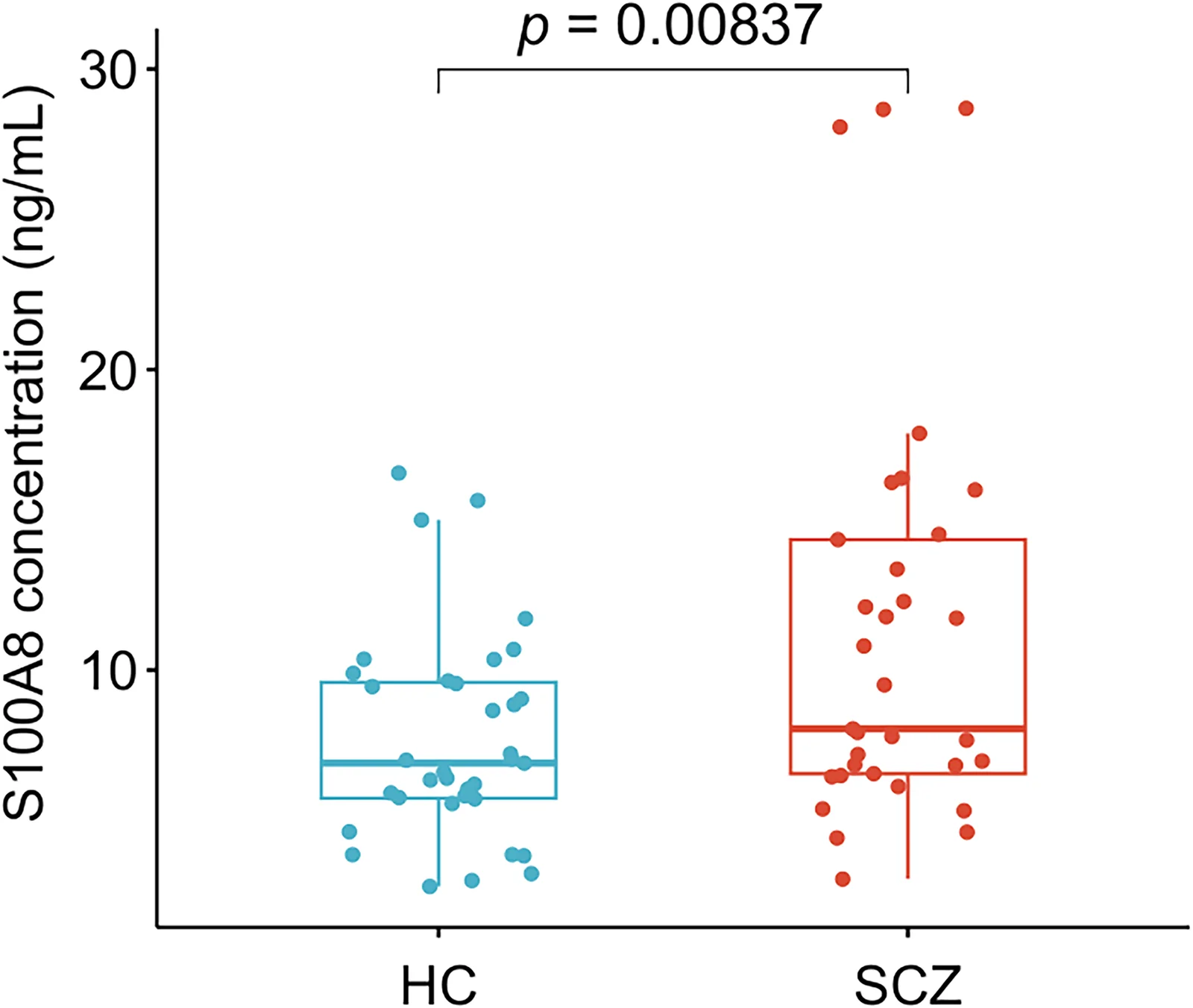
Plasma S100A8 quantification and correlation analysis. Plasma S100A8 protein concentrations detected by ELISA. The SCZ group exhibited significantly higher levels than healthy controls (P < 0.05).

A total of 33 patients with SCZ had complete S100A8, PANSS, age, and sex data. The cohort included 14 men and 19 women, and the median age was 37 years (interquartile range, 31-51). No significant correlation was observed between plasma S100A8 and age (rho = 0.089, P = 0.621), and plasma S100A8 did not differ between men and women (Mann-Whitney U = 129, P = 0.899). For the Wallwork five factors, the Spearman correlation coefficients ranged from -0.199 to -0.016, and none showed a significant correlation after false discovery rate correction (all q = 0.927; **Supplementary Figure S8**; **Supplementary Table S8**). The disorganized/cognitive factor had the largest absolute coefficient (rho = -0.199, 95% CI -0.527 to 0.148, permutation P = 0.267). After adjustment for age and sex, the partial correlation coefficients ranged from -0.220 to 0.012, and none of the five factors was significantly associated with plasma S100A8 (all q = 0.951). The results showed that plasma S100A8 was not related to current PANSS symptom severity. Collectively, these findings support that S100A8 may serve as a peripheral biomarker for disease state rather than symptom severity, and this conclusion requires further verification in larger sample sizes.

## 4 Discussion

SCZ remains a major global health burden, with rising prevalence (40) and 2.52-fold elevated mortality versus controls (41). Limited diagnostics and therapies highlight the need for better mechanistic insights. Emerging evidence links ubiquitination—a key post-translational modification regulating protein stability, localization, and activity—to SCZ pathophysiology through neuronal remodeling and synaptic plasticity (42). This study explores ubiquitin-related genes in SCZ pathogenesis, leveraging the ubiquitin-proteasome system’s role in neurodevelopment. By analyzing ubiquitination-associated genetic signatures, we identify potential biomarkers for early detection and therapeutic targeting. Our findings address critical gaps in understanding protein degradation dysregulation in SCZ, informing strategies for personalized interventions and preventive approaches.

We used an integrated approach to analysis three GEO datasets, which increased the reliability of the results and reduces the bias introduced by a single dataset. In this study, we identified 13 ubiquitination-related differentially expressed genes (URDEGs: *UBR5*, *KPNB1*, *KDM5A*, *BAG3*, *PALLD*, *MLF1*, *S100A8*, *NTRK2*, *STOM*, *ETV5*, *FOXK2*, *PAK2* and *ZFR*) and constructed a molecular signature comprising eight genes (*KPNB1*, *UBR5*, *MLF1*, *S100A8*, *ETV5*, *PAK2*, *KDM5A*, and *ZFR*). Moreover, immune infiltration analysis revealed associations between certain immune cells and these signature genes. Collectively, these results provide insights into SCZ mechanisms and suggest candidate molecular signatures for molecular discrimination.

These identified 13 URDEGs showed significant statistical differences between the SCZ group and the control group, among which 11 genes were extremely statistically significant, and the other two (*PAK2* and *ZFR*) were highly statistically significant. All the 13 URDEGs, as participants in ubiquitination regulation, might be involved in the process of various neurological disorders, including schizophrenia, through the ubiquitin-proteasome pathway according to literature reports. Among the 13 URDEGs, three genes—*UBR5*, *KPNB1*, and *S100A8*—are particularly noteworthy given their central roles in ubiquitination, nuclear transport, and immune regulation, respectively.

Firstly, *UBR5* (ubiquitin protein ligase E3 component n-recognin 5) is a key component of the N-end rule ubiquitination pathway, responsible for recognizing substrates with destabilizing N-terminal residues and mediating their degradation (43). *UBR5* has been implicated in autism spectrum disorders (44), linked to the antidepressant escitalopram treatment response (45), and associated with the regulation of mutant huntingtin and antioxidant mechanisms, influencing the early-onset symptoms of Huntington disease (HD) (46), a mechanism increasingly implicated in SCZ. In our study, *UBR5* expression was significantly upregulated in SCZ brain tissues, suggesting that dysregulation of the ubiquitin-proteasome system may contribute to SCZ pathogenesis. This finding is consistent with prior studies linking *UBR5* to neuropsychiatric disorders (44).

Secondly, *KPNB1* (karyopherin subunit beta 1) mediates nucleocytoplasmic transport through the nuclear pore complex. Defects in nuclear transport have been implicated in various neurological disorders, including SCZ (47). Dysregulation of *KPNB1* may disrupt the nuclear localization of transcription factors and signaling molecules, thereby altering gene expression networks (48). Variants in *KPNB1* have been associated with neurodegenerative conditions like Amyotrophic Lateral Sclerosis (49). Although previous studies have revealed that the rs11871606 variant in *KPNB1* is not linked to the onset of SCZ (50), altered expression of this gene may still contribute to the development of the disorder. In our study, *KPNB1* was upregulated in SCZ and exhibited the highest contribution to the molecular signature (as shown by the nomogram), suggesting its potential utility as a candidate biomarker.

*S100A8* is a calcium-binding protein predominantly expressed in neutrophils and monocytes, in which it participates in innate immune responses and inflammation (51, 52). Its role in SCZ pathogenesis remains controversial: whereas some studies have reported elevated *S100A8* levels in SCZ (52, 53), others have observed no significant difference (54). Our integrated analysis of three independent datasets revealed that *S100A8* was consistently upregulated in SCZ brains and exhibited the strongest positive correlation with myeloid-derived suppressor cells (MDSC) among all signature genes (r = 0.708, P < 0.05). These convergent findings support the neuroinflammation hypothesis of SCZ and suggests that *S100A8* may contribute to immune dysregulation in SCZ potentially by modulating MDSC recruitment. Furthermore, ELISA quantification of plasma S100A8 in an independent cohort of 33 SCZ patients and 35 healthy controls revealed higher protein levels in patients in SCZ patients compared with healthy controls, consistent with the brain tissue mRNA alterations. Within the patient cohort, plasma S100A8 was not associated with age or any of the five PANSS factors and did not differ between men and women. Adjustment for age and sex did not change these results. Together with the higher plasma S100A8 level observed in patients with SCZ than in healthy controls, these findings suggest that S100A8 is a disease-associated peripheral biomarker, but it does not reflect current symptom severity. Notably, antipsychotic drugs such as clozapine have been shown to exert immunomodulatory effects (55), and *S100A8* may serve as a link between inflammation and treatment response.

The remaining 10 URDEGs (*KDM5A*, *BAG3*, *PALLD*, *MLF1*, *NTRK2*, *STOM*, *PAK2*, *ZFR*, *ETV5*, and *FOXK2*) have also been implicated with SCZ or related neurobiological processes, albeit with varying levels of supporting evidence. For instance, *KDM5A* functions as a histone demethylase and has been linked to epilepsy, intellectual disability, and SCZ (56, 57). Similarly, *BAG3* participates in protein quality control and autophagy, with its expression reportedly elevated in specific brain regions of patients with SCZ (58). Collectively, these genes, together with the three aforementioned core genes, underscore the multifaceted nature of ubiquitination dysregulation in SCZ.

GO enrichment analysis revealed that the 13 URDEGs were significantly enriched in biological processes including nuclear protein import and axonogenesis; cellular components such as stress fibers and actomyosin; and molecular functions such as molecular carrier activity and small GTPase binding. These results suggest that URDEGs may contribute to SCZ pathogenesis by modulating nuclear transport and cytoskeletal dynamics. Disruptions in nuclear protein transport can alter gene expression patterns and impair neuronal function (59). Such defects may synergize with dysregulated small GTPase activity—particularly RhoA signaling, which regulates synaptic plasticity and neurotransmitter dynamics via cytoskeletal remodeling (60), to contribute to SCZ-related neurodevelopmental abnormalities. Genetic variants in RhoA-associated pathways further implicate this mechanism in disease susceptibility, while preclinical evidence suggests Rho-kinase inhibitors alleviate cognitive deficits independent of genetic background (61), highlighting their therapeutic potential. Small GTPases, particularly RhoA signaling, have been implicated in synaptic plasticity and may represent potential therapeutic targets for SCZ (61). These findings support a dual therapeutic strategy: pharmacological modulation of GTPase activity to restore nuclear transport efficiency and neuronal morphology, combined with precision medicine approaches leveraging genetic profiling of transport/GTPase pathway variants. Future research should prioritize developing small-molecule regulators targeting RhoA signaling nodes, mapping pathway crosstalk through multi-omics integration, and validating candidates in patient-derived neuronal models. By bridging molecular insights with clinical translation, such efforts could address the heterogeneity of SCZ through mechanism-based therapies tailored to individual genetic and pathophysiological profiles, ultimately improving functional outcomes and quality of life.

Immune profiling revealed significant differences in six immune cell populations between SCZ and control cohorts, notably CD56bright natural killer cells and central memory CD4+ T cells. Risk-stratified analyses further demonstrated distinct immune infiltration patterns across high-/low-risk groups. These findings align with previous studies suggesting that immune system dysfunction plays a key role in SCZ (62, 63), highlighting potential therapeutic targets for immunotherapy development. Emerging studies identify specific immune abnormalities—including altered CD56bright NK cells, follicular helper T cells, and dendritic cells—as candidate biomarkers for SCZ risk prediction (64, 65). While current antipsychotics lack direct immunomodulatory mechanisms, certain agents exhibit indirect immune effects: clozapine demonstrates microglia-specific immunomodulation, while neuroactive steroids and NSAIDs may alleviate symptoms through anti-inflammatory pathways (55). Immunosuppressants and biologics also show therapeutic potential via systemic immune modulation (66). Complementary approaches, particularly traditional Chinese medicine, also offers potential advantages in regulating the immune system (67). Despite progress, critical gaps persist. Future investigations should integrate multi-modal data—neuroimaging, cerebrospinal fluid biomarkers, and preclinical models—to systematically characterize neuroimmune interactions in SCZ. Prioritizing longitudinal studies of immune cell dynamics across disease stages could clarify causal mechanisms, while combinatorial strategies targeting both neuronal and immune pathways may yield synergistic therapeutic effects. This dual focus on mechanistic exploration and translational validation will advance precision immunopsychiatry for SCZ management.

Unlike S100A9 (68) and S100A8 (69) models prioritizing generalized immune transcripts, or proteasome-subunit studies describing global UPS downregulation (70), our analysis restricted feature selection to curated ubiquitination-related genes and derived an eight-gene LASSO signature (KPNB1, UBR5, MLF1, S100A8, ETV5, PAK2, KDM5A, ZFR) with moderate discriminatory capacity (AUC 0.71–0.89) for stratifying SCZ patients into high-and low-risk groups. Combined with independent plasma S100A8 ELISA validation and network analyses revealing PPI connections among five signature genes and key regulatory nodes such as CEBPB, this ubiquitin-first framework bridges brain UPS candidacy to a peripherally measurable inflammatory marker, offering a complementary diagnostic tool and testable hypotheses for future non-invasive biomarker development.

Several limitations of this study should be acknowledged. First, the clinical cohort used for ELISA validation was small and medication status was not controlled, which may confound peripheral S100A8 levels; moreover, S100A8 is a non-specific inflammatory marker and should not be interpreted as specific to SCZ. Validation in larger, medication-naïve first-episode cohorts with longitudinal sampling is required. Second, only peripheral plasma protein validation was performed in clinical samples; additional cellular and animal experiments are still required to clarify the causal roles of these genes in SCZ pathogenesis. Third, the 8-gene diagnostic model was developed and evaluated within a merged cohort of three GEO datasets (GSE21138, GSE21935 and GSE12649, n = 88 SZ and 82 controls), i.e., internal validation; although the protein level of S100A8 was further verified in an independent clinical cohort, external validation of the entire model in an independent dataset is still lacking and is recognized as the primary direction of future work. Finally, despite efforts to mitigate batch effects, potential inter-batch variation across multiple datasets remains a concern. These issues will be addressed in our future investigations.

## 5 Conclusions

In conclusion, this study conducted a comprehensive analysis of differentially expressed ubiquitination-related genes in SCZ by integrating multiple GEO datasets, and subsequently developed and validated a novel molecular signature. The findings offer important insights for advancing the understanding of the mechanisms underlying SCZ and provide a theoretical basis for the development of novel non-invasive biomarkers. Future research could benefit from expanding the sample size and performing in-depth validation through *in vivo* or *in vitro* experiments to enhance the reliability of the conclusions. Specifically, larger, prospectively designed studies incorporating medication-naïve first-episode patients, longitudinal sampling, and appropriate disease controls, as well as external validation of the gene signature in independent cohorts, are warranted.

## Data Availability

The datasets presented in this study can be found in online repositories. The names of the repository/repositories and accession number(s) can be found in the article/**Supplementary Material**.
