# Supplementary material for: Integrated bioinformatics analysis identifies a ubiquitination-related gene signature in schizophrenia with peripheral clinical validation
Source: Front Psychiatry. 2026 Oct 29;17:1987536. doi: 10.3389/fpsyt.2026.1987536 (PMC13754837; doi:10.3389/fpsyt.2026.1987536)
Supplement: Supplementary file 1 [file SupplementaryFile1.doc]

**Supplemental Figures:**

**Figure S1.** Flow Chart for the Comprehensive Analysis of URDEGs. **Figure S2.** Data processing. **Figure S3.** GO Enrichment Analysis for URDEGs. **Figure S4.** GSEA for Combined Datasets. **Figure S5.** Differential Gene Expression Analysis and GSEA for Risk Group. **Figure S6.** PPI Network and Regulatory Network of Signature Genes. **Figure S7.** Risk Group Immune Infiltration Analysis by ssGSEA Algorithm. **Figure S8.** Correlations between plasma S100A8 and Wallwork PANSS five-factor scores. **Table S1**. GEO Microarray Chip Information. **Table S2.** URDEGs. **Table S3.** Result of GO and KEGG Enrichment Analysis for URDEGs. **Table S4**. Results of GSEA for Combined Datasets. **Table S5.** Results of GSEA for Risk Group. **Table S6.** 16 TFs of the constructed PPI Network. **Table S7.** Patient characteristics and univariate associations of age and sex with plasma S100A8 and PANSS factors. **Table S8.** Unadjusted and age- and sex-adjusted associations between plasma S100A8 and Wallwork PANSS five-factor scores.

**Fig. S1 Flow Chart for the Comprehensive Analysis of URDEGs**

SCZ, Schizophrenia; GSEA, Gene Set Enrichment Analysis; DEGs, Differentially Expressed Genes; URGs, Ubiquitin-Related Genes; GO, Gene Ontology; KEGG, Kyoto Encyclopedia of Genes and Genomes; URDEGs, Ubiquitin-Related Differentially Expressed Genes. ROC, Receiver Operating Characteristic; PPI, Protein-protein Interaction; ssGSEA, single-sample Gene-Set Enrichment Analysis; TF, Transcription Factor.

**Fig. S2 Batch Effects Removal of GSE21138, GSE21935 and GSE12649**

A. before going to batch processing integrated GEO data setdistribution box plot. B. Post-batch integrated GEO Datasets distribution boxplots. C. PCA plot of the datasets before debatching. D. Go to the PCA map of the Combined GEO Datasets after batch processing. PCA, Principal Component Analysis. Yellow GSE21138 for SCZ dataset, green for SCZ GSE21935 data sets, blue GSE12649 for SCZ data set.

**Fig. S3 GO Enrichment Analysis for URDEGs**

A. URDEGs enrichment of GO histogram analysis results show: BP, CC, MF. Abscissa to GO terms. B-D. URDEGs related to GO enrichment of network diagram analysis results show: BP (B), CC (C), the MF (D). Orange nodes represent entries, green nodes represent molecules, attachment on behalf of the entry and molecular relationship. Enrichment of GO analysis was based on the selection criteria of the p value < 0.05.

**Fig. S4 GSEA for Combined Datasets**

A. Integrated GEO data setset of GSEA bubble chart shows four biological function. B-E. GSEA showed a significant enrichment in all genes in Oxidative Stress Response Up (B), Lysosome (C), Energy Dependent Regulation Of MTOR By Lkb1 Ampk (D), Clathrin Mediated Endocytosis (E). Enrichment of bubble size in the bubble chart represents the number of genes, bubble color represents the size of the NES value, the deeper the red color, the greater the NES the color blue NES is smaller. GSEA selection criteria for the value *P*< 0.05.

**Fig. S5 Differential Gene Expression Analysis and GSEA for Risk Group**

A. Volcano plot of differentially expressed genes between high-risk and low-risk groups in SCZ samples from the integrated GEO dataset. B. Heatmap of the expression values of the top 10 upregulated and top 10 downregulated differentially expressed genes ranked by logFC in high-risk and low-risk groups in SCZ samples. C.Bubble chart displaying GSEA results for four biological functions in SCZ samples. D-G. GSEA showed that the high risk of SCZ was significantly enriched in Oxidative Stress Response Up (D), Oxidative Stress By Hne And Tbh (E), and oxidative stress by HNE and TBH (E). Oxidative Stress Response (F) and Pi3kakt Signaling Pathway (G). Heat maps grouped in shallow red stands for High Risk group, light blue represents the Low Risk group. Red represents high expression in heat map, blue represents lower expression. Enrichment of bubble size in the bubble chart represents the number of genes, bubble color represents the size of the NES value, the deeper the red color, the greater the NES the color blue NES is smaller. GSEA selection criteria for the value p < 0.05.

**Fig. S6 PPI Network and Regulatory Network of Model Genes**

A. The PPI Network of model genes calculated by the STRING database. B. mRNA-miRNA Regulatory Network of Model Genes. C. mRNA-TF Regulatory Network of Model Genes. Blue as the mRNA, green as micrornas, orange for TF. PPI: Protein-Protein Interaction Network.

**Fig. S7 Risk Group Immune Infiltration Analysis by ssGSEA Algorithm**

A.Comparison of immune cells in the HighRisk group and LowRisk group of SCZ samples. B- C. Low risk of SCZ samples group (B) and high risk (C) the correlation between the immune cells heat maps. D-E. low risk of SCZ samples group (D) and high risk (E) the immune cells infiltrating abundance Genes with the Model Genes the relevance of the bubble chart. Ns on behalf of the p value of 0.05 or higher, no statistical significance; * represents the value < 0.05, p was statistically significant on behalf of *** p value < 0.001, highly statistically significant. Correlation coefficient (r value) absolute value below 0.3 as weak or irrelevant, as the weak correlation between 0.3 - 0.5, for moderate correlation between 0.5 - 0.8. Shallow blue to be low risk group, light red for the high-risk group. Red is the positive correlation, blue is the negative correlation. The depth of the color represents the strength of the correlation.

**Fig. S8 Correlations between plasma S100A8 and Wallwork PANSS five-factor scores**

Circles represent unadjusted Spearman correlations, and squares represent partial Spearman correlations adjusted for age and sex. Horizontal lines represent 95% confidence intervals.

**Supplemental Tables:**

**Table S1 GEO Microarray Chip Information**

|  | GSE21138 | GSE21935 | GSE12649 |
| --- | --- | --- | --- |
| Platform | GPL570 | GPL570 | GPL96 |
| Species | Homo sapiens | Homo sapiens | Homo sapiens |
| Tissue | Brain | Brain | Brain |
| Samples in SCZ group | 30 | 23 | 35 |
| Samples in Control group | 29 | 19 | 34 |
| Reference | PMID: 30016334 | PMID: 30016334 | PMID: 28732597 |

GEO, Gene Expression Omnibus; SCZ, Schizophrenia.

**Table S2 URDEGs**

UBR5

KPNB1

KDM5A

BAG3

PALLD

MLF1

PAK2

S100A8

NTRK2

ZFR

STOM

ETV5

FOXK2

**Table S3 Result of GO and KEGG Enrichment Analysis for URDEGs**

| ONTOLOGY | ID | Description | GeneRatio | BgRatio | pvalue |
| --- | --- | --- | --- | --- | --- |
| BP | GO:0006606 | protein import into nucleus | 3/12 | 159/18800 | 1.23 e-04 |
| BP | GO:0051170 | import into nucleus | 3/12 | 163/18800 | 1.33 e-04 |
| BP | GO:0017038 | protein import | 3/12 | 210/18800 | 2.81 e-04 |
| BP | GO:0051169 | nuclear transport | 3/12 | 308/18800 | 8.58 e-04 |
| BP | GO:0007409 | axonogenesis | 3/12 | 430/18800 | 2.24 e-03 |
| CC | GO:0001725 | stress fiber | 2/13 | 69/19594 | 9.30 e-04 |
| CC | GO:0042641 | actomyosin | 2/13 | 77/19594 | 1.16 e-03 |
| CC | GO:0032432 | actin filament bundle | 2/13 | 78/19594 | 1.19 e-03 |
| CC | GO:0030018 | Z disc | 2/13 | 127/19594 | 3.10 e-03 |
| CC | GO:0031674 | I band | 2/13 | 139/19594 | 3.70 e-03 |
| MF | GO:0140104 | molecular carrier activity | 2/13 | 73/18410 | 1.18 e-03 |
| MF | GO:0031267 | small GTPase binding | 2/13 | 267/18410 | 1.47 e-02 |
| MF | GO:0051020 | GTPase binding | 2/13 | 298/18410 | 1.81 e-02 |
| MF | GO:0045296 | cadherin binding | 2/13 | 333/18410 | 2.23 e-02 |
| MF | GO:0004712 | protein serine/threonine/tyrosine kinase activity | 2/13 | 446/18410 | 3.83 e-02 |

GO, Gene Ontology; BP, Biological Process; CC, Cellular Component; MF, Molecular Function; URDEGs, Ubiquitin-Related Differentially Expressed Genes.

**Table S4 Results of GSEA for Combined Datasets**

| ID | Set Size | Enrichment Score | NES | p value |
| --- | --- | --- | --- | --- |
| ELVIDGE_HYPOXIA_BY_DMOG_UP | 98 | 0.56310 | 2.25645 | 1.79 e-05 |
| ELVIDGE_HYPOXIA_UP | 126 | 0.54018 | 2.25145 | 1.76 e-05 |
| GROSS_HYPOXIA_VIA_ELK3_AND_HIF1A_UP | 86 | 0.54346 | 2.13175 | 1.80 e-05 |
| GROSS_HYPOXIA_VIA_ELK3_DN | 104 | 0.49266 | 1.99229 | 1.78 e-05 |
| MANALO_HYPOXIA_UP | 162 | 0.45896 | 1.98113 | 1.74 e-05 |
| MCMURRAY_TP53_HRAS_COOPERATION_RESPONSE_UP | 16 | 0.70844 | 1.91816 | 1.09 e-03 |
| WEIGEL_OXIDATIVE_STRESS_BY_HNE_AND_TBH | 45 | 0.54028 | 1.87406 | 5.92 e-04 |
| MENSE_HYPOXIA_UP | 62 | 0.50242 | 1.85678 | 2.57 e-04 |
| ONGUSAHA_TP53_TARGETS | 26 | 0.59629 | 1.82712 | 2.41 e-03 |
| NEMETH_INFLAMMATORY_RESPONSE_LPS_UP | 61 | 0.48778 | 1.79713 | 7.71 e-04 |
| XU_HGF_SIGNALING_NOT_VIA_AKT1_6HR | 20 | 0.62520 | 1.79595 | 4.26 e-03 |
| FARDIN_HYPOXIA_11 | 16 | 0.65760 | 1.78050 | 5.16 e-03 |
| CHUANG_OXIDATIVE_STRESS_RESPONSE_UP | 20 | 0.61725 | 1.77311 | 5.05 e-03 |
| JAIN_NFKB_SIGNALING | 50 | 0.40055 | 1.46740 | 2.98 e-02 |
| REACTOME_CLATHRIN_MEDIATED_ENDOCYTOSIS | 90 | 0.36126 | 1.48079 | 1.28 e-02 |
| KEGG_LYSOSOME | 78 | 0.39020 | 1.55895 | 7.25 e-03 |
| MANALO_HYPOXIA_DN | 185 | 0.35364 | 1.61802 | 3.37 e-04 |
| REACTOME_ENERGY_DEPENDENT_REGULATION_OF_MTOR_BY_LKB1_AMPK | 17 | 0.57850 | 1.63581 | 1.90 e-02 |
| ELVIDGE_HYPOXIA_BY_DMOG_DN | 43 | 0.48577 | 1.72363 | 3.53 e-03 |
| ELVIDGE_HYPOXIA_DN | 112 | 0.51835 | 2.20499 | 2.29 e-05 |

GSEA, Gene Set Enrichment Analysis.

**Table S5 Results of GSEA for Risk Group**

| ID | Set Size | Enrichment Score | NES | p value |
| --- | --- | --- | --- | --- |
| GROSS_HYPOXIA_VIA_ELK3_DN | 104 | 0.53136639 | 2.22240 | 2.05 e-05 |
| FULCHER_INFLAMMATORY_RESPONSE_LECTIN_VS_LPS_DN | 286 | 0.45359836 | 2.17403 | 2.08 e-05 |
| ELVIDGE_HYPOXIA_BY_DMOG_UP | 98 | 0.52378009 | 2.16966 | 2.05 e-05 |
| GROSS_HYPOXIA_VIA_ELK3_AND_HIF1A_UP | 86 | 0.53052935 | 2.15094 | 2.05 e-05 |
| ELVIDGE_HYPOXIA_UP | 126 | 0.47441764 | 2.04541 | 2.06 e-05 |
| CHUANG_OXIDATIVE_STRESS_RESPONSE_UP | 20 | 0.69285716 | 2.04121 | 1.21 e-04 |
| SEKI_INFLAMMATORY_RESPONSE_LPS_UP | 46 | 0.55554126 | 1.99636 | 2.02 e-05 |
| ONGUSAHA_TP53_TARGETS | 26 | 0.62071952 | 1.95550 | 4.24 e-04 |
| WEIGEL_OXIDATIVE_STRESS_BY_HNE_AND_TBH | 45 | 0.54088273 | 1.93340 | 2.22 e-04 |
| WP_OXIDATIVE_STRESS_RESPONSE | 25 | 0.47454945 | 1.48095 | 4.58 e-02 |
| WP_NOTCH_SIGNALING | 31 | 0.45049252 | 1.47975 | 3.94 e-02 |
| WP_PI3KAKT_SIGNALING_PATHWAY | 238 | 0.26670199 | 1.25271 | 4.46 e-02 |
| MANALO_HYPOXIA_DN | 185 | 0.32992974 | 1.49344 | 3.10 e-03 |
| XU_HGF_TARGETS_REPRESSED_BY_AKT1_DN | 74 | 0.40781761 | 1.60116 | 5.19 e-03 |
| RAHMAN_TP53_TARGETS_PHOSPHORYLATED | 11 | 0.69505563 | 1.73601 | 7.81 e-03 |
| MODY_HIPPOCAMPUS_POSTNATAL | 33 | 0.52834151 | 1.75815 | 3.41 e-03 |
| HINATA_NFKB_TARGETS_KERATINOCYTE_DN | 12 | 0.71969038 | 1.84097 | 2.40 e-03 |
| ELVIDGE_HYPOXIA_BY_DMOG_DN | 43 | 0.53973503 | 1.90480 | 3.75 e-04 |
| WP_HIPPOCAMPAL_SYNAPTOGENESIS_AND_NEUROGENESIS | 26 | 0.62145557 | 1.95428 | 4.16 e-04 |
| ELVIDGE_HYPOXIA_DN | 112 | 0.50090511 | 2.11023 | 1.95 e-05 |

GSEA, Gene Set Enrichment Analysis.

**Table S6 mRNA-TF**

| **mRNA** | **TF** |
| --- | --- |
| KPNB1 | AR |
| KPNB1 | CEBPB |
| KPNB1 | FOXA1 |
| KPNB1 | FOXA2 |
| KPNB1 | NRF1 |
| KPNB1 | SPI1 |
| KDM5A | EP300 |
| KDM5A | ERG |
| KDM5A | FOS |
| KDM5A | POLR2A |
| KDM5A | CEBPB |
| KPNB1 | MYC |
| PAK2 | MYC |
| S100A8 | MAX |
| S100A8 | RUNX1 |
| S100A8 | USF1 |
| UBR5 | YY1 |
| UBR5 | CEBPB |
| UBR5 | CTCF |
| UBR5 | MAX |
| UBR5 | MYC |
| ZFR | NRF1 |
| ZFR | YY1 |

**Table S7 Patient characteristics and univariate associations of age and sex with plasma S100A8 and PANSS factors.**

Panel A. Patient characteristics

| Characteristic | Value |
| --- | --- |
| Patients with complete data | 33 |
| Age, years, median (IQR) | 37 (31-51) |
| Men, n (%) | 14 (42.4) |
| Women, n (%) | 19 (57.6) |
| Plasma S100A8, median (IQR) | 8.046 (6.554-14.342) |

Panel B. Univariate covariate associations

| Outcome | Age, rho | Age P | Sex, rank-biserial r | Sex P |
| --- | --- | --- | --- | --- |
| Plasma S100A8 | 0.089 | 0.621 | -0.030 | 0.899 |
| Positive factor | -0.024 | 0.894 | 0.199 | 0.340 |
| Negative factor | 0.012 | 0.949 | -0.015 | 0.956 |
| Disorganized/cognitive factor | 0.119 | 0.508 | -0.128 | 0.544 |
| Excited factor | -0.089 | 0.623 | -0.147 | 0.487 |
| Depressed factor | -0.121 | 0.504 | 0.312 | 0.111 |

Table S8 Unadjusted and age- and sex-adjusted associations between plasma S100A8 and Wallwork PANSS five-factor scores.

| PANSS factor | Items | Unadjusted rho (95% CI) | Permutation P | FDR q | Adjusted partial rho (95% CI) | Adjusted P | Adjusted FDR q |
| --- | --- | --- | --- | --- | --- | --- | --- |
| Positive | P1, P3, P5, G9 | -0.051 (-0.398 to 0.281) | 0.781 | 0.927 | -0.034 (-0.384 to 0.324) | 0.856 | 0.951 |
| Negative | N1, N2, N3, N4, N6, G7 | -0.122 (-0.435 to 0.218) | 0.497 | 0.927 | -0.123 (-0.458 to 0.242) | 0.509 | 0.951 |
| Disorganized/cognitive | P2, N5, G11 | -0.199 (-0.527 to 0.148) | 0.267 | 0.927 | -0.220 (-0.533 to 0.145) | 0.234 | 0.951 |
| Excited | P4, P7, G8, G14 | -0.035 (-0.407 to 0.327) | 0.848 | 0.927 | -0.032 (-0.382 to 0.326) | 0.863 | 0.951 |
| Depressed | G2, G3, G6 | -0.016 (-0.397 to 0.403) | 0.927 | 0.927 | 0.012 (-0.344 to 0.364) | 0.951 | 0.951 |
